# Spatial epidemiology of acute respiratory infections in children under 5 years and associated risk factors in India: District-level analysis of health, household, and environmental datasets

**DOI:** 10.3389/fpubh.2022.906248

**Published:** 2022-12-13

**Authors:** Karuppusamy Balasubramani, Kumar Arun Prasad, Naveen Kumar Kodali, Nishadh Kalladath Abdul Rasheed, Savitha Chellappan, Devojit Kumar Sarma, Manoj Kumar, Rashi Dixit, Meenu Mariya James, Sujit Kumar Behera, Sulochana Shekhar, Praveen Balabaskaran Nina

**Affiliations:** ^1^Department of Geography, Central University of Tamil Nadu, Thiruvarur, Tamil Nadu, India; ^2^Department of Epidemiology and Public Health, Central University of Tamil Nadu, Thiruvarur, Tamil Nadu, India; ^3^IGAD Climate Prediction and Applications Centre (ICPAC), Nairobi, Kenya; ^4^Department of Public Health and Community Medicine, ICMR—National Institute of Traditional Medicine, Belgaum, Karnataka, India; ^5^Department of Molecular Biology, ICMR—National Institute for Research in Environmental Health, Bhopal, Madhya Pradesh, India; ^6^Department of Microbiology, ICMR—National Institute for Research in Environmental Health, Bhopal, Madhya Pradesh, India; ^7^Department of Public Health and Community Medicine, Central University of Kerala, Kasaragod, Kerala, India

**Keywords:** ARI, PM_2.5_ and PM_10_, unclean cooking fuel, Getis-Ord Gi^*^ spatial statistic, NFHS-4

## Abstract

**Background:**

In India, acute respiratory infections (ARIs) are a leading cause of mortality in children under 5 years. Mapping the hotspots of ARIs and the associated risk factors can help understand their association at the district level across India.

**Methods:**

Data on ARIs in children under 5 years and household variables (unclean fuel, improved sanitation, mean maternal BMI, mean household size, mean number of children, median months of breastfeeding the children, percentage of poor households, diarrhea in children, low birth weight, tobacco use, and immunization status of children) were obtained from the National Family Health Survey-4. Surface and ground-monitored PM_2.5_ and PM_10_ datasets were collected from the Global Estimates and National Ambient Air Quality Monitoring Programme. Population density and illiteracy data were extracted from the Census of India. The geographic information system was used for mapping, and ARI hotspots were identified using the Getis-Ord Gi^*^ spatial statistic. The quasi-Poisson regression model was used to estimate the association between ARI and household, children, maternal, environmental, and demographic factors.

**Results:**

Acute respiratory infections hotspots were predominantly seen in the north Indian states/UTs of Uttar Pradesh, Bihar, Delhi, Haryana, Punjab, and Chandigarh, and also in the border districts of Uttarakhand, Himachal Pradesh, and Jammu and Kashmir. There is a substantial overlap among PM_2.5_, PM_10_, population density, tobacco smoking, and unclean fuel use with hotspots of ARI. The quasi-Poisson regression analysis showed that PM_2.5_, illiteracy levels, diarrhea in children, and maternal body mass index were associated with ARI.

**Conclusion:**

To decrease ARI in children, urgent interventions are required to reduce the levels of PM_2.5_ and PM_10_ (major environmental pollutants) in the hotspot districts. Furthermore, improving sanitation, literacy levels, using clean cooking fuel, and curbing indoor smoking may minimize the risk of ARI in children.

## Introduction

Respiratory infections are one of the major causes of chronic diseases in children worldwide, causing significant deaths in developing countries and greatly contributing to increases in disability-adjusted life years (1, 2). Globally, lower respiratory infections (LRI) are a major cause of death in children under 5 years (3), and in 2015, an estimated 104.8 children per million died of LRI (2). In India, even though there was a substantial decrease in the deaths of children under 5 years (2.516 million deaths in 2000 to 1.201 million deaths in 2015), the count is still the highest in the world in this category (4). Among children under 5 years in India, pneumonia—a major manifestation of LRI—was the second leading cause of mortality, with 0.191 million deaths (15.9%), next to preterm birth complications (0.33 million, 27.5%) (5).

Etiology and risk factors for respiratory tract infections are multifactorial: solid fuel cookstove use, household tobacco use, and agricultural crop burning are important environmental risk factors for respiratory tract infections in children in India (6–8). Solid fuel is a key risk factor for acute respiratory infections (ARI) (6, 9–13), and 50% of pneumonia-related deaths in children under 5 years are due to inhalation of particulate matter (soot) arising from indoor air pollution (14). When inhaled, particulate matter (PM_2.5_) can settle deep in the lungs and can enter into circulation, resulting in an increased risk of respiratory and cardiac illnesses (15). Solid fuel burning, windblown soil, vehicular exhaust, agricultural crop burning, and fine particulate matter (PM_2.5_) from construction sites pollute air and cause respiratory diseases (16). Improved sanitation and hygiene interventions have been reported to reduce ARI in children (17, 18).

The National Family Health Survey-4 (NFHS-4) estimated the prevalence of ARI to be 2.7% in 2015–2016 in India, which is a ~50% decrease from 5.6% reported in 2005–2006 by the NFHS-3 (19). Despite a ~50% decrease in ARI mortality of children under 5 years (4), India has not achieved the target of reducing child mortality by two-thirds between 1990 and 2015, as envisaged in the Millennium Development Goal 4 (20). Achieving the sustainable development goal target of child survival (neonatal mortality of ≤12/1,000 live births and mortality of children under 5 years of ≤25/1,000 live births) by 2030 (21) may not be possible for many states in India at the current rate of progress (5).

As ARIs are major determinants of child mortality, understanding their spatial distribution at the district level across India is critical for devising targeted intervention strategies. In this study, we have mapped the prevalence of ARI in children under 5 years at the district level across India using data from the NFHS-4 and the Census of India. In addition, using spatial statistics, we have mapped the hotspots to identify high-risk districts and states in India. In addition, the spatial association of environmental (PM_2.5_ and PM_10_), household (cooking fuel, sanitation, and vaccination status), and behavioral (tobacco smoking) risk factors with ARI epidemiology in children under 5 years at the district level across India was also outlined. Furthermore, the statistical association of ARI and its associated risk factors were estimated using the quasi-Poisson regression model.

## Methods

### Data source

Data on ARI, unclean fuel, improved sanitation, mean maternal BMI, mean household size, mean number of children, median months of breastfeeding the children, percentage of poor households, percentage of children with diarrhea, percentage of low birth weight, percentage of people who smoke tobacco, and immunization status of children data were obtained from the NFHS-4, a district-level survey carried out in all Indian districts for determining many important health indicators, including ARI (22). The survey was carried out across India from January 2015 to December 2016. The NFHS-4 data are available for public use at https://dhsprogram.com/ and can be used after obtaining authorization from the Demographic and Health Survey (DHS) program. Data on the district-wise population (total and children) and illiteracy data (2011) for all 640 districts in India were collected from the Census of India website (https://www.censusindia.gov.in/2011census/dchb/DCHB.html) for mapping and comparative analysis. The average concentration of PM_2.5_ for districts for the period 2013–2016 was collected from the Global Estimates data. The Global Estimates of fine particulate matter concentration are collated by combining data retrieved from aerosol optical depth (AOD) from satellite products, GEOS-Chem chemical transport modeling on surface PM_2.5_, and ground-monitored data. The satellite products, such as NASA's MODIS C6.1, MISR V23, NASA's MAI ACC6 and SeaWiFS, were used to derive AOD. The estimates were calibrated with the global ground-based PM_2.5_ observations to obtain long-term consistency for trend assessment. The year-wise net CDF files were downloaded from the Global Estimates V4.GL.03/V4.GL.03.NoGWR datasets available at https://sites.wustl.edu/acag/datasets/surface-pm2-5/. In addition to PM_2.5_ data, ground-monitored PM_10_ data from 168 cities in India were collected from the National Ambient Air Quality Monitoring Programme (23) for the period 2013–2016 for comparative analysis. The datasets used for analysis are provided in Supplementary material S1.

### Study measures

#### Outcome variable

The outcome of interest is the number of ARI cases per 100 population in each of the 640 districts in India. ARI cases are identified based on symptoms of (a) cough accompanied by (b) short, rapid breathing that is chest-related and/or (c) difficult breathing that is chest-related. A child with all these three symptoms was considered to be suffering from ARI (22).

#### Independent variables

The particulate matter, PM_2.5_ (average annual concentrations of PM_2.5_ in μg/m^3^), was used as an environmental variable in the analysis. The following variables were considered household variables: (a) unclean cooking fuel use (percentage of households using solid fuels including coal, charcoal, wood, straw/shrub/grass, and animal dung), (b) improved sanitation facilities (percentage of households using non-shared toilet type with piped sewer systems), and (c) tobacco consumption (percentage of people smoking tobacco). Mothers' characteristics include median months of breastfeeding, the mean number of children, and mean maternal BMI. The percentage of children with diarrhea, low birth weight, and immunization status of children (percentage of <2-year-old children immunized with BCG, measles, polio, and DPT) were considered children-level variables. The demography variables included (a) mean household size, (b) rural/urban residence (dominant category of residence in a district), (c) illiteracy level (percentage of illiterates to the total population), and (d) percentage of poor households.

### Data normalization and mapping

In this study, the data points of PM_2.5_ with 0.01° × 0.01° resolution from each district boundary across India were spatially averaged (all the pixel values within a district boundary) to derive the annual PM_2.5_ data. To avoid temporal deviations and compare the PM_2.5_ values with NFHS-4 ARI datasets, a 4-year (2013–2016) average concentration of PM_2.5_ was computed. Similarly, the 4-year values of location-specific PM_10_ data were averaged and compared with PM_2.5_ through the GIS.

All the generated datasets were prepared as spatial layers for spatial analysis and mapped using ArcMap 10.4 software (https://www.esri.com/en-us/arcgis/products/arcgis-desktop/resources). We used choropleth mapping techniques for better visualization and easy interpretation.

### Hotspot analysis

Spatial autocorrelation investigates the degree to which a phenomenon is correlated to itself in a two-dimensional geographical surface. The evaluation of such identifiable spatial patterns of ARI is necessary to better understand the propagation of ARI in a geographical area. The overall clustering of the incidence of ARI can be measured by global spatial autocorrelation methods. For example, Moran's I statistic tests the null hypothesis that the spatial autocorrelation of a variable is assumed to be zero. The variable is said to have spatial autocorrelation if the null hypothesis is rejected (24). A family of G statistics, developed by Getis and Ord, is more popular and is used to study spatial interdependency. The general G statistic (25), similar to Moran's I statistics, measures only the overall degree of spatial autocorrelation and results in a single index for the entire study area. However, the global statistic is too general, and the local patterns in spatial autocorrelation are neutralized and will go undetected (26, 27). Local-level spatial autocorrelation methods will focus on factors controlling the local variation in a larger space, and the detection of such clusters will enable us to identify hotspots without any preconceptions about their locations (28). Getis and Ord proposed G_i_ and ${\text{G}}_{\text{i}}^{*}$, a local spatial autocorrelation statistic more suitable for detecting significant pockets of clustering, which are often undetected using global statistics (29).

In this study, we used district-level NFHS-4 data on the percentage of children with ARI symptoms for detecting ARI clusters in India. The hotspot analysis tool in ArcGIS software was used to identify the intensity of clustering in a bin (district) relative to its neighboring bins (districts). The hotspot analysis was performed by determining the standardized Getis-Ord Gi^*^ spatial statistic with the null hypothesis that there is no spatial autocorrelation among the features with respect to the variable ARI cases (in %). The analysis revealed the identification of ARI hotspots based on Z-scores (standard deviations) and *p*-values (probability). The standardized Getis-Ord Gi^*^ spatial statistic considering *w*_*ii*_ ≠ 0 can be expressed as follows:


$$\begin{array}{l} G_{i}^{*}\left(d\right)=\frac{\sum_{j}w_{ij}\left(d\right)x_{j}-W_{i}^{*}\bar{x}}{s{\left\{\left[\left(nS_{1i}^{*}\right)-W_{i}^{*2}\right]/\left(n-1\right)\right\}}^{1}/2},\text{ all }j \end{array}$$

where $W_{i}^{*}=W_{i}+w_{ii};\;S_{1i}^{*}=\sum_{j}w_{ij}^{2}\left(\text{all}\;j\right)$; *w*_*ij*_ is the spatial weight between the features *i* and *j*; $\bar{x}$ and *s* represent the sample mean and standard deviation, respectively; *n* represents the total number of features; and *Wi* represents the sum of weights for a distance *d*, and that can be expressed as $W_{i}=\sum_{j\neq i}w_{ij}(d)$.

The Gi^*^ statistic generates the *p*-value obtained from the significance test, and that could be used to determine whether or not to accept the null hypothesis. The standardized G (Z-score of G) can be calculated as follows (26):


$$\begin{array}{l} Z_{G}=\frac{\left(G-E\left(G\right)\right)}{\sqrt{V\left(G\right)}} \end{array}$$

where E(G) represents the expected G and V(G) is the variance of G. The E(G) and V(G) can be calculated as follows:


$$\begin{array}{l} E\left(G\right)=\frac{\overset{n}{\underset{i=1}{\sum }}\overset{n}{\underset{j=1}{\sum }}W_{ij}\left(d\right)}{n\left(n-1\right)},\;\forall_{j}\neq i\\ \;V\left(G\right)=E\left(G^{2}\right)-E{\left(G\right)}^{2} \end{array}$$

When the absolute value of the Z-score is large, and the *p* < 0.05, the null hypothesis can be rejected, that is, the districts (or ARI values associated with the districts) exhibit statistically significant clustering.

In this study, the local sum of ARI cases (in %) of a single district and its neighboring districts is compared proportionally with the sum of ARI cases (in %) of all Indian districts. We have defined a Euclidean distance of 200 km as a spatial limit to define the neighborhood relationships. Thus, the prevalence of ARI in each of the districts is compared spatially with the neighboring districts (the districts that are within 200 km radial distance) as well as with all the 640 districts of India. As a result of this comparison, a Z-score (standard deviation) and a *p*-value (probability) are generated for each district within the context of the neighboring districts at 95% CI. When the local sum is quite different from the expected local sum, and that difference is larger than expected to be from random chance, a statistically significant Z-score (*p* < 0.05) is obtained (30). A high Z-score and a small *p*-value for a district indicate a spatial clustering of high values (hotspots). A low negative Z-score and a small *p*-value indicate a spatial clustering of low values (cold spots). A Z-score near zero indicates no apparent spatial clustering (31).

### Statistical analysis

A quasi-Poisson regression model was used to estimate the association between ARI (cases per 100 population) and the independent variables (PM_2.5_, percentage of unclean fuel, percentage of improved sanitation, percentage of childhood immunization, percentage of illiteracy level, percentage of people smoking tobacco, mean maternal BMI, mean household size, mean number of children, median months of breastfeeding, percentage of poor household, percentage of children with diarrhea, percentage of low birth weight, and rural/urban residence). The quasi-Poisson regression model was used to account for the overdistribution in the count data. All the independent variables were included in the multivariate analysis, irrespective of significance in the univariate analysis. There was no multicollinearity issue among the independent variables. The adjusted incidence rate ratios (aIRRs) and the corresponding 95% CI are presented. A *p* < 0.05 was considered significant. The statistical analysis was carried out using STATA software version 17 (StataCorp, TX, USA).

## Results

### Spatial pattern of ARI

**Figure 1:**
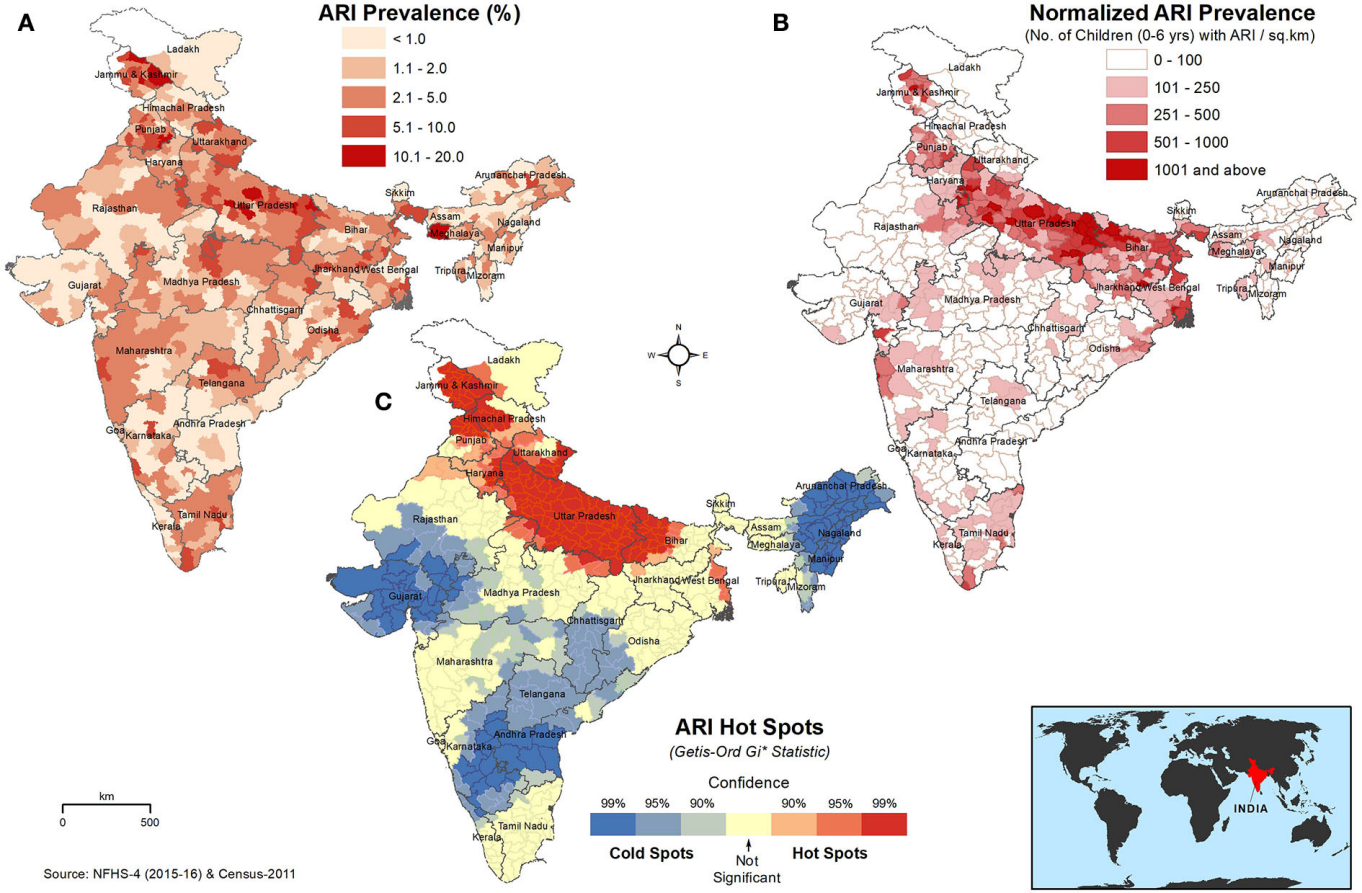
Spatial distribution of **(A)** absolute percentage of ARI in children under 5 years, **(B)** ARI prevalence normalized to the children population density (children with ARI/km^2^), and **(C)** hot and cold spots of ARI in children under 5 years. The darker red shades indicate higher values/hotspots of ARI. The inset map shows the location of India on the world map.

The prevalence of ARI in children under 5 years in each district was taken as an absolute percentage of the population, as shown in Figure 1A. The average percentage of children (under 5 years) having symptoms of ARI in India during the study period was 2.7% (6,529 out of 238,945 surveyed children). The district-wise ARI percentage is classified into five classes: <1.0- very low; 1.1-2.0-low; 2.1-5.0-moderate; 5.1-10.0-high; 10.1-20.0- very high. The class interval of 10.1–20.0 (%) represents districts that have the highest prevalence rates, while the class interval of <1.0 shows districts with a very low prevalence of ARI. Many districts in the northern states of India, such as Jammu and Kashmir, Punjab, Uttarakhand, and Uttar Pradesh, show the prevalence of ARI prevalence of more than 5%. In addition, a few districts in Bihar, West Bengal, Assam, Meghalaya, Jharkhand, Odisha, and Madhya Pradesh report a prevalence of ARI of >5%. In the districts surrounding the major cities of Delhi, Kolkata, Mumbai, Bhopal, and Hyderabad, the prevalence of ARI was >5%. Many districts in the northern states were found to have the prevalence of ARI of >5%, with several other districts varying between 2–5%. Similarly, many districts of Maharashtra (west), Telangana (south), and Tamil Nadu (south) showcased the prevalence of ARI between 2–5%.

The absolute ARI prevalence in percentage was normalized to the children population density at the district level for relative comparison, as shown in Figure 1B. The highest prevalence of ARI (>1,001 cases/km^2^) was observed predominantly in the districts of the Great Plains of India (Indus–Ganga–Brahmaputra plains), which stretches from Jammu and Kashmir to West Bengal and includes the states of Punjab, Haryana, Uttar Pradesh, and Bihar. Several districts in Uttar Pradesh showed highest prevalence rates of ARI among children. The highest incidence of ARI per km^2^ was also observed in Kolkata, Mumbai, Delhi, Chennai, Hyderabad, and Chandigarh, which are major cities in India.

The hotspots of ARI in children under 5 years are shown in Figure 1C. ARI hotspots, with a 95% confidence level, were predominantly seen in north India, covering the entire state/UTs of Uttar Pradesh and Delhi and many districts of Bihar, Haryana, Punjab, Uttarakhand, Himachal Pradesh, and Jammu and Kashmir.

### Spatial distribution of environmental pollutants and population density

**Figure 2:**
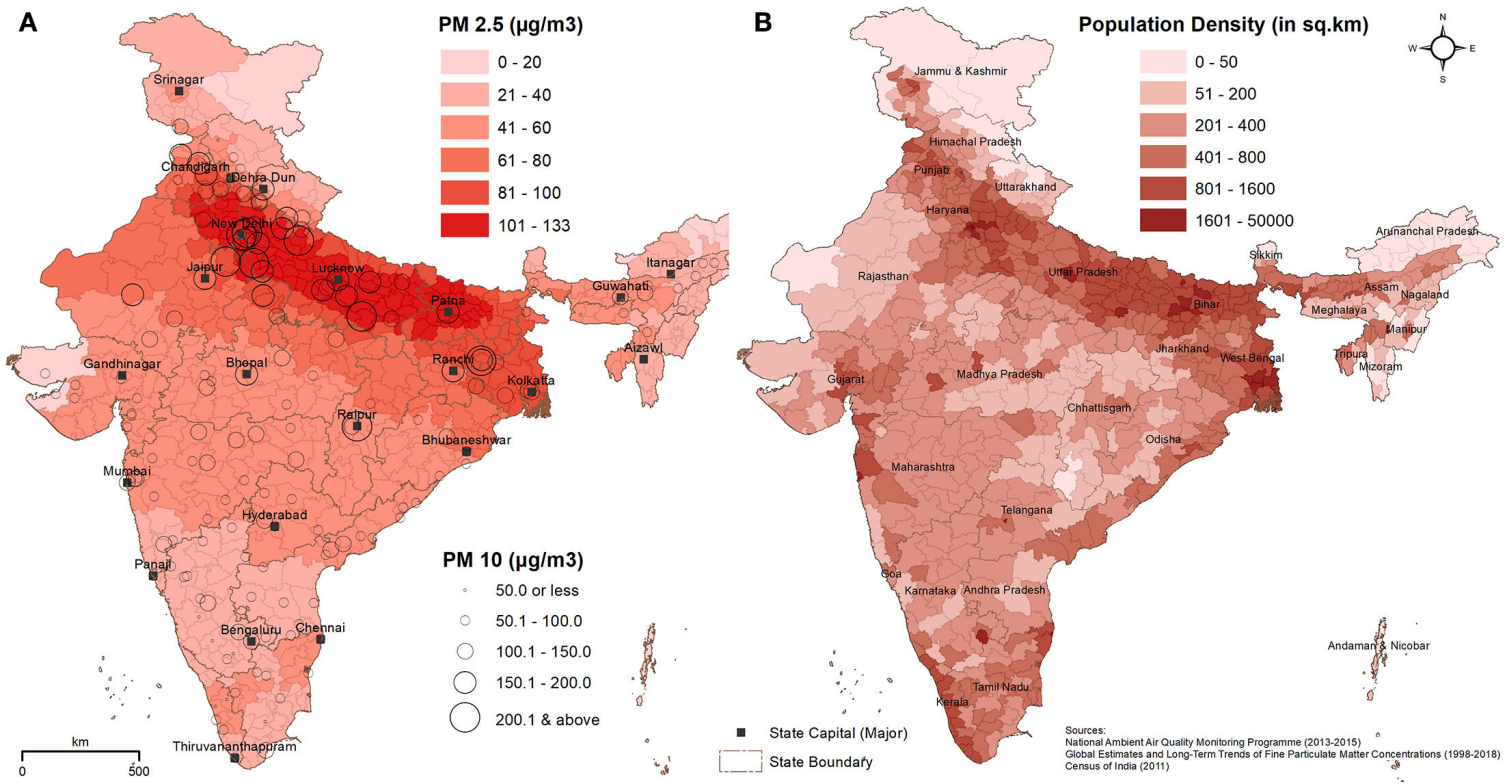
Spatial distribution of **(A)** ambient air pollutants PM_2.5_ (μg/m^3^) and PM_10_ (μg/m^3^), and **(B)** population density (persons/km^2^). The darker red and brown shades in the maps denote higher PM_2.5_ and population density, respectively. The higher concentrations of PM_10_ in the major cities of India are represented by larger circles.

The ambient PM_2.5_ (μg/m^3^) and PM_10_ (μg/m^3^) concentrations at the district level across India are shown in Figure 2A. The average district-wise PM_2.5_ concentration in India was 62.4 μg/m^3^. The entire floodplains of the Ganges River in north India were gravely affected by high concentrations of PM_2.5_. In several districts of Uttar Pradesh, Haryana, Delhi, and Bihar, alarming concentrations (>100 μg/m^3^) of PM_2.5_ were observed. PM_2.5_ concentrations surrounding the Ganges Plains were also very high (60–80 μg/m^3^). The average PM_10_ concentration in Indian cities was 104.1 μg/m^3^. The highest concentrations of PM_10_ (>200 μg/m^3^) were observed in the cities of the Ganges Plains, especially around the National Capital Territory (NCT) of Delhi. The majority of the developed cities in the states of Punjab, Haryana, Uttar Pradesh, Uttarakhand, Bihar, Jharkhand, and West Bengal were the major hotspots for PM_10_. The state capitals of Chhattisgarh (Raipur), Madhya Pradesh (Bhopal), and Rajasthan (Jaipur) also reported alarming concentrations (150–200 μg/m^3^) of PM_10_.

The density of the population (persons/km^2^) at the district level is shown in Figure 2B. In addition to megacities of India, such as Mumbai, Kolkata, Delhi, Chennai, Bengaluru, and Hyderabad, the most populous districts are found in the states of Uttar Pradesh, Punjab, Bihar, Jammu and Kashmir, and West Bengal, with a density of >800 persons per km^2^.

### Spatial distribution of significant household, children, and demography factors

**Figure 3:**
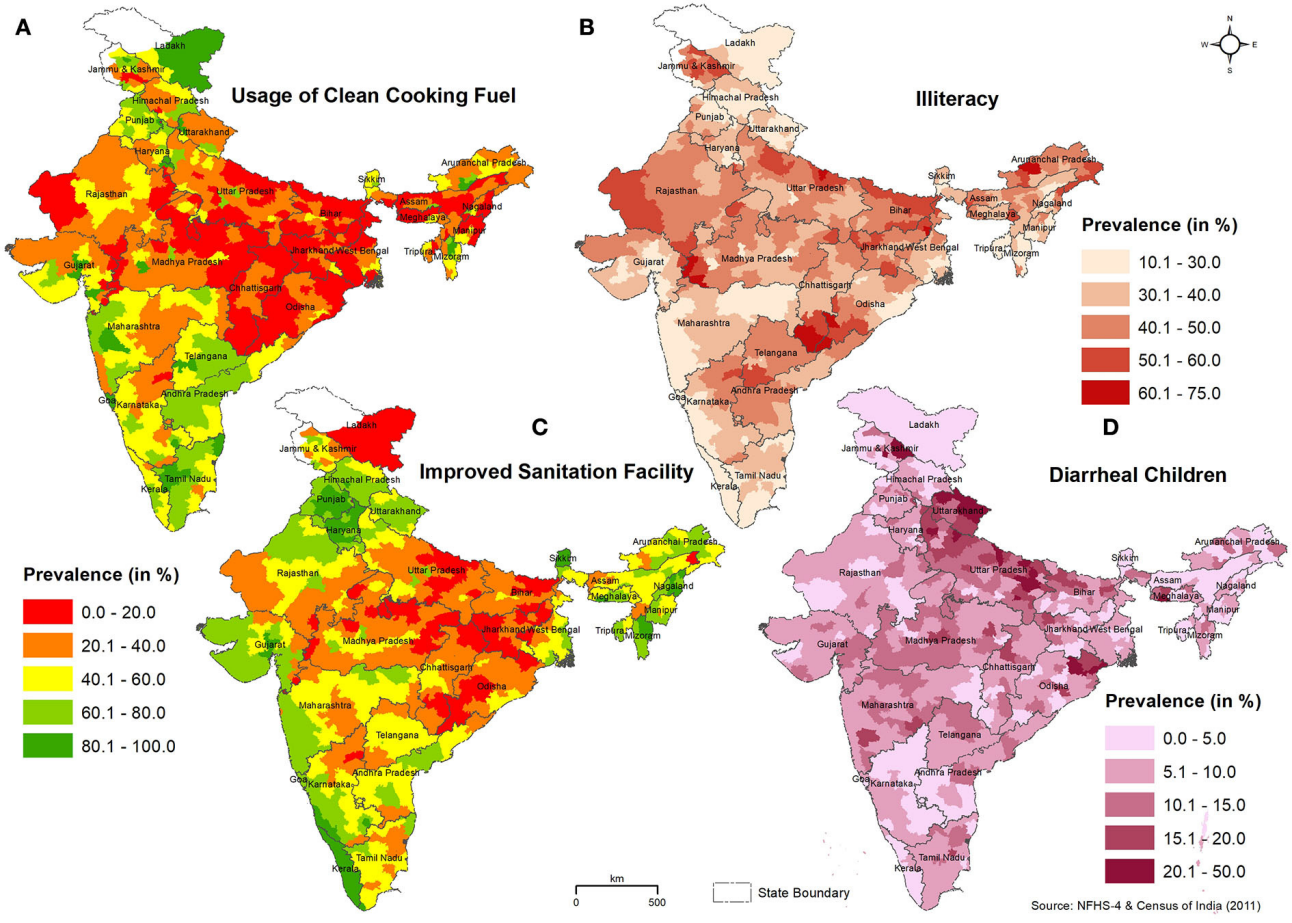
Spatial pattern of **(A)** usage of clean cooking fuel, **(B)** illiteracy rate, **(C)** improved sanitation facility, and **(D)** children with diarrhea in India. The district-wise percentage value of each parameter is classified into five classes.

The district-wise pan-India distribution of significant household, children, and demographic factors is shown in Figures 3A–D. The usage of clean cooking fuel across different districts of India is shown in Figure 3A. Clean fuel includes the use of electricity, LPG/natural gas, or biogas in the household. The district-level data showed that the mean usage of clean fuel for cooking was 37.5%. The class interval of 0.0–20 (%) indicated the districts with the lowest usage of clean fuel, and these districts were predominantly in the north and eastern parts of India, comprising the states of Uttar Pradesh, Madhya Pradesh, Chhattisgarh, Bihar, Jharkhand, Odisha, West Bengal, Assam, and Meghalaya. Clean fuel usage was high in many districts of South India, especially in the states of Tamil Nadu, Kerala, Andhra Pradesh, and Maharashtra.

The spatial pattern of the illiteracy rate is shown in Figure 3B. The Census of India (2011) data showed that the average illiteracy rate in Indian districts was 37.6%. Except for Kerala and certain districts in Mizoram, the illiteracy rate across districts in India is more than 20%. Most of the districts in the northern states (Uttar Pradesh, Bihar, Rajasthan, Madhya Pradesh, and Jharkhand) have a higher illiteracy rate (>40%). The improved sanitation facility (non-shared toilet facilities in the households) at the district level across India is shown in Figure 3C. The average availability of improved sanitation facilities in Indian districts was 47.7%. Many districts in the ARI-prevalent states of Uttar Pradesh, Bihar, Jharkhand, Madhya Pradesh, and Odisha had a low prevalence of sanitation facilities, while states such as Kerala, Punjab, and Haryana had improved sanitation facilities. The prevalence of diarrhea among children in India was comparatively highest in several districts of Uttar Pradesh, Uttarakhand, and Bihar states and a few districts in the states of Jammu and Kashmir, Jharkhand, Meghalaya, Madhya Pradesh, and Maharashtra (Figure 3D).

### Spatial distribution of smoking, a behavioral risk factor

**Figure 4:**
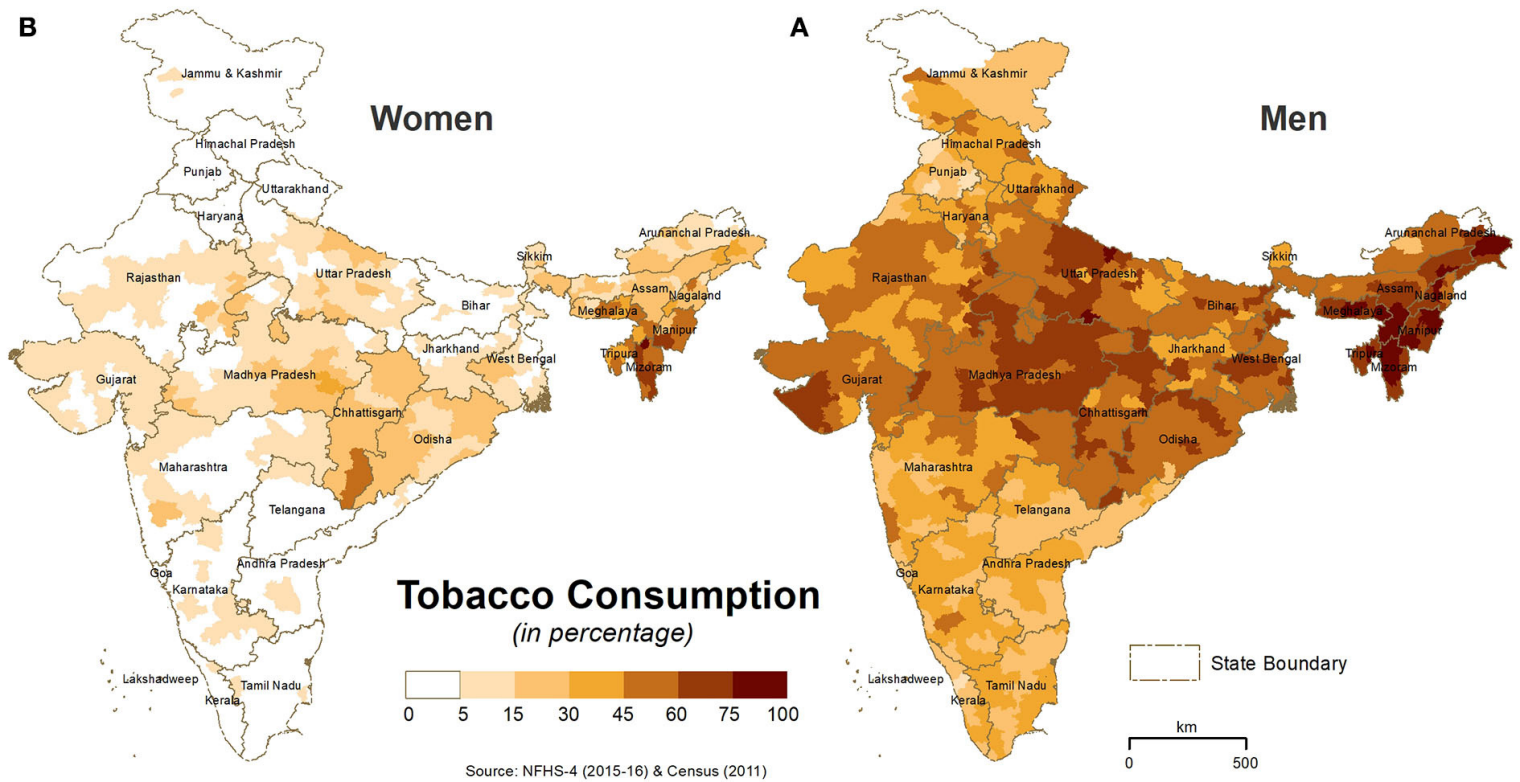
District-wise spatial distribution of **(A)** tobacco consumption by men and **(B)** tobacco consumption by women in India. The darker shades in the maps show higher tobacco consumption.

The district-wise spatial distribution (%) of tobacco consumption among men and women is shown in Figure 4, and the state-wise distribution (%) of cigarettes and bidis, the two dominant types of smoking tobacco across the different states/UTs in India, is shown in Supplementary Figure S1. In addition to the northern-eastern (NE) states, the prevalence (>60%) of tobacco consumption by men was high in a majority of districts of Madhya Pradesh and Uttar Pradesh (Figure 4A). Tobacco consumption by women was >5% in the districts of the northern states of India (Figure 4B). Even though the national average of smoking/tobacco use in India was around 13%, the ARI-dominated northern (Jammu and Kashmir, Himachal Pradesh, Uttarakhand, and Haryana) and central (Madhya Pradesh) states showed >20% tobacco consumption (either in cigarettes or bidis, or both). All the NE states and West Bengal also reported a very high prevalence of smoking/tobacco consumption.

### Distribution of risk factors in the ARI hotspots

The average distribution of risk factors in the major ARI hotspot states/UT of India is given in Table 1. The normalized ARI cases (ARI children per sq.km) in the hotspot states/UTs of Uttar Pradesh, Bihar, and Delhi were higher than average cases in all Indian districts. Delhi was 20 times higher than the national average. The average levels of environmental pollutants (PM_2.5_ and/ PM_10_) were higher in all the hotspot states, and in Delhi, it was more than two times the national average. The household variables such as clean cooking fuel, improved sanitation facilities, and status of children with diarrhea were worse in Bihar and Uttar Pradesh. When compared with the national average, men's tobacco consumption is also high in Bihar and Uttar Pradesh.

**Table 1:** Distribution of environmental, household, demographic, and behavioral variables in the ARI hotspot states/UTs of India.

**State/UT**	**Normalized ARI (ARI Children/ km^2^)**	**PM_2.5_ (μg/m^3^)**	**PM_10_ (μg/m^3^)**	**Clean cooking fuel (%)**	**Illiteracy (%)**	**Improved sanitation (%)**	**Diarrhoeal children (%)**	**Men's tobacco consumption (%)**	**Women's tobacco consumption (%)**
***National***	***336***	***62.4***	***104.1***	***37.4***	***37.5***	***47.6***	***8.4***	***47.8***	***9.6***
	**(0–42,236)**	***(0–113.2)***	***(31.5-261.9)***	***(0–99.5)***	***(11.3–71.2)***	***(0–99.5)***	***(0–44.7)***	***(0–100)***	***(0–78.1)***
Bihar	549	102.7	160.4^*^	16.4	49.6	24.8	9.8	50.8	2.9
	(96–2,147)	(72.3–124.2)		(5.2–51.7)	(39.2–59.2)	(12.5–49.9)	(3.1–18.1)	(36.1–63.6)	(0.4–7.6)
Uttar Pradesh	653	109.8	163.7	28.9	42.9	32.3	14.3	54.5	8.0
	(11–2,504)	(62.9–131.3)	(43.5-262.0)	(9.2–78.5)	(29.2–62.1)	(10.4–67.1)	(4.2–29.1)	(34.1–79.2)	(1.6–21.8)
Delhi	6,361	131.1	219.3^*^	97.6	23.4	74.3	9.1	33.3	1.5
	(0–42,236)	(128.3–133.2)		(94.5–99.5)	(19.6–28.1)	(64.6–87.8)	(5.0–18.1)	(18.9–48.2)	(0.8–2.6)
Haryana	300	106.7	134.7	49.4	34.6	79.2	7.5	36.2	1.6
	(0–1,551)	(64.8–131.0)	(101.3-173.0)	(17.2–82.6)	(26.6–58.2)	(46.7–91.0)	(1.6–15.2)	(21.8–57.7)	(0.3–5.7)
Punjab	249	77.8	127.4	63.6	33.6	81.6	7.2	19.4	0.1
	(24–682)	(55.5–102.6)	(73.5-188.3)	(40.6–80.7)	(24.4–45.0)	(74.7–89.2)	(3.4–17.4)	(10.7–35.8)	(0–0.5)

The average value of a state represents the average of all the district values within the particular state, and the national average value indicates the average of all the 640 districts in the country. The values within brackets represent the range of values.

* PM_10_ values were obtained from only one city in these states/UTs. The bold values indicate the national average of the respective variables.

### Statistical analysis of the prevalence of ARI and associated factors

The multivariate association between the prevalence of ARI cases per 100 population and children, mothers, household, and environmental variables estimated by the quasi-Poisson regression model is shown in Table 2. The descriptive statistics of the dependent and independent variables are presented in Supplementary Table S1. There was a statistically significant association between ARI and PM_2.5_ μg/m^3^, percentage of illiterates, the percentage of children with diarrhea, and the mother's mean BMI. For each μg/m^3^ increase in the PM_2.5_ level, the rate of ARI cases increased by 0.6% (aIRR 1.006; 95% CI 1.003–1.008). The rate of ARI increased by 1.1% for each percentage increase in district-level illiteracy (aIRR 1.011; 95% CI 1.002–1.021). For each percentage point increase in diarrhea (aIRR 1.059; 95% CI 1.051–1.068), the rate of ARI increased by 5.9%. The rate of ARI increased by 1.3 times with an increase in each mean maternal BMI (aIRR 1.320, 95% CI: 1.206–1.445). The predictors (unclean fuel use %, improved sanitation %, percentage of poor households, mean household size, and the number of children ever born to a woman) were associated with the prevalence of ARI only in the univariate analysis.

**Table 2:** Association between ARI cases and environmental, household, behavioral, and demographic variables.

**Independent variables**	**uIRR**	**95% CI**	***p***	**aIRR**	**95% CI**	***p***
PM 2.5 (μg/m^3^)	1.007	1.005–1.009	**0.000**	1.006	1.003–1.008	**0.000**
Unclean fuel use (%)	1.004	1.001–1.007	**0.012**	1.004	0.998–1.010	0.200
Improved sanitation (%)	0.995	0.992–0.998	**0.001**	0.999	0.993–1.004	0.580
Illiteracy (%)	1.015	1.008–1.022	**0.000**	1.011	1.002–1.021	**0.022**
Child immunization (%)	1.000	0.997–1.004	0.871	1.001	0.997–1.005	0.657
Rural districts vs. urban districts	1.245	1.067–1.454	**0.005**	1.109	0.917–1.342	0.288
Smoking tobacco men (%)	1.001	0.997–1.005	0.672	1.002	0.996–1.008	0.536
Smoking tobacco women (%)	0.992	0.987–0.997	**0.003**	0.999	0.992–1.007	0.852
Diarrhea in children (%)	1.068	1.060–1.077	**0.000**	1.059	1.051–1.068	**0.000**
Low birth weight (%)	0.991	0.979–1.002	0.121	1.006	0.993–1.019	0.354
Poor households (%)	1.003	1.001–1.006	**0.008**	1.004	0.997–1.011	0.244
Maternal BMI (mean)	1.035	0.981–1.091	0.211	1.320	1.206–1.445	**0.000**
Household size (mean)	1.195	1.104–1.294	**0.000**	1.027	0.931–1.133	0.597
Number of children (mean)	1.394	1.207–1.611	**0.000**	0.810	0.629–1.044	0.103
Months of breastfeeding (median)	1.012	0.988–1.036	0.331	1.005	0.981–1.030	0.668

uIRR, unadjusted incidence rate ratio; aIRR, adjusted incidence rate ratio; BMI, body mass index. The bold values indicate that the values are significant at *p* < 0.05.

## Discussion

The two primary outcomes of the study were (i) mapping the spatial distribution and hotspots of ARI at the district level and (ii) mapping the environmental, household, demography, and behavioral risk factors across India to find their association with ARI. The hotspot districts of ARI cases were predominantly from the states/UTs of Uttar Pradesh, Bihar, Delhi, Haryana, and Punjab (Indo-Gangetic Plain inhabitants). Spatial mapping showed PM_2.5_, PM_10_, population density, tobacco use, clean fuel, and improved sanitation facilities to have a strong overlap with ARI, while statistical analysis showed PM_2.5_, population density, and improved sanitation facilities to have a significant association with ARI among children under 5 years in India.

The states of Uttar Pradesh, Punjab, Bihar, and West Bengal were densely populated (>800 persons/sq. km) and had a high incidence of ARI. The heavily populated megacities of Kolkata, Delhi, Mumbai, Hyderabad, and Chandigarh also showed a high incidence of ARI, suggesting a strong link between population density, urbanization, and ARI. Similar findings were reported in several studies conducted in India, where overcrowding was associated with the prevalence of ARI in children under 5 years (32, 33). Many megacities in India are densely populated and comprise many slums; Dharavi in Mumbai is the biggest slum in Asia. Slums characterized by poor urban populations, overcrowded with unhygienic conditions, and congested housing are favorable hotspots for many respiratory diseases (34). In Brazil, a high incidence of ARI has been reported in children from slums (34, 35). Furthermore, the high transmission of viruses in slums is facilitated by resource-limited facilities, lack of appropriate spacing, improper sanitation systems, poor nutritional status, and education (36).

Spatial mapping indicated strong overlap between environmental pollutants (PM_2.5_ and PM_10_) and ARI incidence in children. In this study, the entire Great Plains of India comprising the Indus, Ganges, and Brahmaputra floodplains in the north, covering the states/UTs of Punjab, Haryana, Delhi, Uttar Pradesh, and Bihar, showed alarming concentrations (>100 μg/m^3^) of PM_2.5_ and (>150 μg/m^3^) PM_10_. Importantly, these states/UTs also showed a very high incidence of ARI, indicating a strong link between PM_2.5_, PM_10_, and ARI. The risk of ARI was suggested to increase by 12%; for every 10 μgm, there was a 3 μg/m^3^ rise in PM_2.5_ (37). The quasi-Poisson regression model based on district-level data showed a 1.7% rise in the number of ARI cases for each additional unit increase in PM_2.5_ levels. Vehicular and industrial pollution, agricultural crop burning, solid fuel cookstove use, and household tobacco use were the primary sources of PM_2.5_ and were associated with increased hospitalization related to adverse respiratory conditions (16). The highly productive and fertile Indo-Gangetic basin supports more than 200 million lives and hosts >10% of India's coal-fired power generation plants. The entire region has high levels of nitrogen and sulfur oxides, which lead to increased particulate matter suspensions in the air and respiratory diseases (38). Agricultural crop residue burning (ACRB) in northern India (Haryana and Punjab) was a major contributor to PM_2.5_, and the downwind spiked PM_2.5_ levels in Delhi to ~20 times the permissible concentration specified by the WHO (8). In areas where ACRB is intense, there was a three-fold increase in ARI, and children under 5 years were the most vulnerable (8). The increased levels of PM_2.5_ had a significant association with population density (39). Spatial analysis indicated that the heavily populated states, particularly in the Indo-Gangetic plains, to have high concentrations (>100 μg/m^3^) of PM_2.5_, suggesting the strong interplay between population density and increased PM_2.5_ levels in increasing the prevalence of ARI among children under 5 years in India.

Diarrhea is significantly associated with ARI in children under 5 years. Acute lower respiratory tract infections (ALRIs) and diarrhea are the major causes of morbidity and mortality among children under 5 years (40). A study conducted among infants aged 0–23 months in Pakistan reported children comorbid with diarrhea to have higher odds of ARI (41). A quantitative analysis among Nepali and Indian children found the incidence of ARI to increase when an episode of diarrhea occurred within 28 days before the onset of ARI (40). Similarly, in Ghana, within 2–4 weeks of the occurrence of diarrhea, the risk of ALRI substantially increased among the children (42). A previous study showed that diarrhea led to acute loss of micronutrients, dehydration, and stresses in the immune system, resulting in the increased risk of ALRI (42). The body mass index of the mother was also associated with the increased risk of ARI among children under 5 years in India. Similar observations were reported by Rajappan et al. (43), where high BMI of the mother was associated with increased risks of wheezing, prolonged cough, and lower respiratory tract infection in their children. A significant association between pre-pregnancy maternal obesity and asthma and current wheezing in 7–8 years children was reported in a development study cohort in Amsterdam (44). Maternal obesity was found to affect the development of the lower airway tract in infants, and the alterations in airway branching may result in an increased risk of respiratory infections (43).

A strong spatial association between the usage of unclean cooking fuel and the hotspots of ARI was observed. The results from the univariate analysis also showed a significant association between the prevalence of ARI and unclean fuel use. Household air pollution (HAP), majorly contributed by the use of coal, wood, charcoal, animal dung, and crop residues for cooking and heating, was identified as a major cause of respiratory illness and death in children under 5 years in India (45). Children under 5 years were found to be highly vulnerable to HAP if they spend a considerable amount of time with their mothers in the kitchen (46, 47). An estimated 3 billion people, especially from low- and middle-income countries (LMICs), cook with solid fuels, which are the primary sources of household air pollution (HAP) (46). In India, in 2017, an estimated 55% of the population used solid fuels for cooking (48). The ARI hotspots in India, clustered primarily in central and northern belts of India, pointed toward the use of unclean cooking fuel in these states. The use of solid fuel was relatively higher (≥80%) in north and central India (Bihar, Uttar Pradesh, Madhya Pradesh, Jharkhand, Chhattisgarh) and some states in the east (Odisha and West Bengal) and the north-east (Assam and Meghalaya). Among the states that used high solid fuel, Uttar Pradesh and Bihar reported a >5% ARI incidence (>10 children/km^2^), while Jharkhand and West Bengal reported a >2% incidence (2–5 children/km^2^). Also, the prevalence of ARI (persons per sq. km) was observed to be relatively higher in these states. A similar trend in the spatial distribution in the increased PM_2.5_ level (101–133 ug/m^3^) was observed in the central and northern belts, especially in New Delhi, Uttar Pradesh, and Bihar. The WHO guideline for 24 hours mean PM_2.5_ is 25 μg/m^3^. However, in India, HAP as high as 609 μg/m^3^ has been recorded in the kitchen of rural households (47). A recent Indian study that utilized NFHS-4 datasets showed that households using unclean cooking fuel, households without separate kitchens, and smoking inside the house increased the likelihood of ARI in children under 5 years presumably due to indoor air pollution (49). Similarly, a prospective case–control study in South India (50) and a prospective observational study in rural central India (6) have documented the association between indoor air pollution and the risk of ARI in children. HAP exposure–response studies in other LMICs, such as Guatemala and Nepal, have also shown a direct association between HAP and ARI (51, 52). In order to promote the use of clean cooking fuel, several initiatives were introduced by the Indian government, including the Pradhan Mantri Ujjwala Yojana scheme, giving free liquid petroleum gas (LPG) connection to over 80 million households below the poverty line (53) and is in the process of replacing solid cooking fuel for cooking with LPG in 80% of the households by 2019 (54).

In most ARI hotspot states, cigarettes and bidis are widely smoked. The use of bidi is very high in the states of West Bengal, Himachal Pradesh, Haryana, Uttarakhand, and Madhya Pradesh, which are also the hotspot states of ARI. Tobacco smoking, contributing majorly to indoor air pollution, is an important risk factor for ARI (55). In a cross-sectional study conducted in India, the analysis of NFHS-4 data indicated that indoor smoking increased ARI susceptibility in children under 5 years (49). Particulate matter is one of the major toxic components found in tobacco smoke. Fractions of PM_10_ and PM_2.5_ deposit and retain in the respiratory tract (56), and long-term exposure to PM_2.5_ increases the risk of ARI in children (57).

This study found most districts, including those with a higher rate of the prevalence of ARI, lack access to improved sanitation facilities. When mapped for sanitation facilities, the ARI hotspot states of Uttar Pradesh, Bihar, and Madhya Pradesh had relatively lower sanitation facilities, while Uttarakhand, Punjab, Himachal Pradesh, and Haryana, and the other ARI hotspots ranked higher in the sanitation levels. A similar observation was made by Reese et al. in their matched cohort study carried out in Odisha, India, which reported no reduction in ARI on improved water and sanitation intervention (58). On the contrary, several randomized controlled trials showed a strong association between hygiene interventions and reduction in ARI symptoms in urban children of LMICs (59). A randomized control trial carried out in Bangladesh showed a reduced prevalence of reported ARI in the household that received sanitation intervention (latrines, potties, scoops, chlorinated drinking water, handwashing alone, or in combination with nutritional supplements) compared with the control group (17). Similarly, the analysis of Demographic Health Survey (DHS) data in Bangladesh showed that a lack of improved water, sanitation, and hygiene facilities was associated with higher odds of ARI in children under 5 years (60). Statistical analysis in this study showed that improved sanitation facilities were associated with a reduction in the rate of ARI, which is in line with previous findings.

This study has its limitations and challenges. Importantly, the study results are not causative and reflect only associations between the independent variables and ARI. The statistical analysis was challenging as the datasets were aggregated from multiple sources (NFHS-4, Census of India, satellite products, and ground-based stations). Few of the independent variables were self-reported, and the respondents may have given answers that are generally desirable, especially regarding cooking fuel and improved sanitation.

## Conclusion

Population density, environmental pollution (PM_2.5_ and PM_10_), comorbidity of diarrhea, maternal BMI, and sanitation were found to be significantly associated with ARI in children under 5 years in India; most of the ARI hotspot states with a higher population density exhibited a relatively higher level of PM_2.5_. Among the household factors, the use of clean cooking fuel, poor sanitation, tobacco consumption, and illiteracy rate shared a strong spatial association with ARI in many hotspot districts, suggesting a synergistic role for multiple variables in causing ARI. Overall, this study provides important insights into the spatial distribution of ARI hotspots and associated risk factors at the district level across India. To draw decisive inferences, more research and modeling studies are required, especially in the hotspot states/UTs, to support policymakers and stakeholders to formulate a comprehensive action plan to reduce ARI in children.

## Data availability statement

Publicly available datasets were analyzed in this study. This data can be found here: https://www.dhsprogram.com.

## Ethics statement

The studies involving human participants were reviewed and approved by Institutional Review Board of International Institute for Population Sciences, Mumbai. Written informed consent to participate in this study was provided by the participants' legal guardian/next of kin.

## Author contributions

KB and PB designed the study and wrote the initial manuscript. KB, NA, and PB collected the data. KB, KP, NK, NA, DS, and PB analyzed the data. KB, NK, SC, DS, MK, RD, MJ, SB, KP, SS, and PB interpreted the data. All authors reviewed and finalized the manuscript.

## Supplementary material

Supplementary Figure S1 State-wise spatial distribution of **(A)** cigarettes and **(B)** bidis (hand-rolled tobacco product) in India. The darker red shades in the maps show higher tobacco consumption.

Supplementary Table S1 Descriptive statistics of the dependent and independent variables.
