# Supplementary figures and images for: Spatial epidemiology of acute respiratory infections in children under 5 years and associated risk factors in India: District-level analysis of health, household, and environmental datasets
Source: Front Public Health. 2022 Dec 13;10:906248. doi: 10.3389/fpubh.2022.906248 (PMC9792853; doi:10.3389/fpubh.2022.906248)

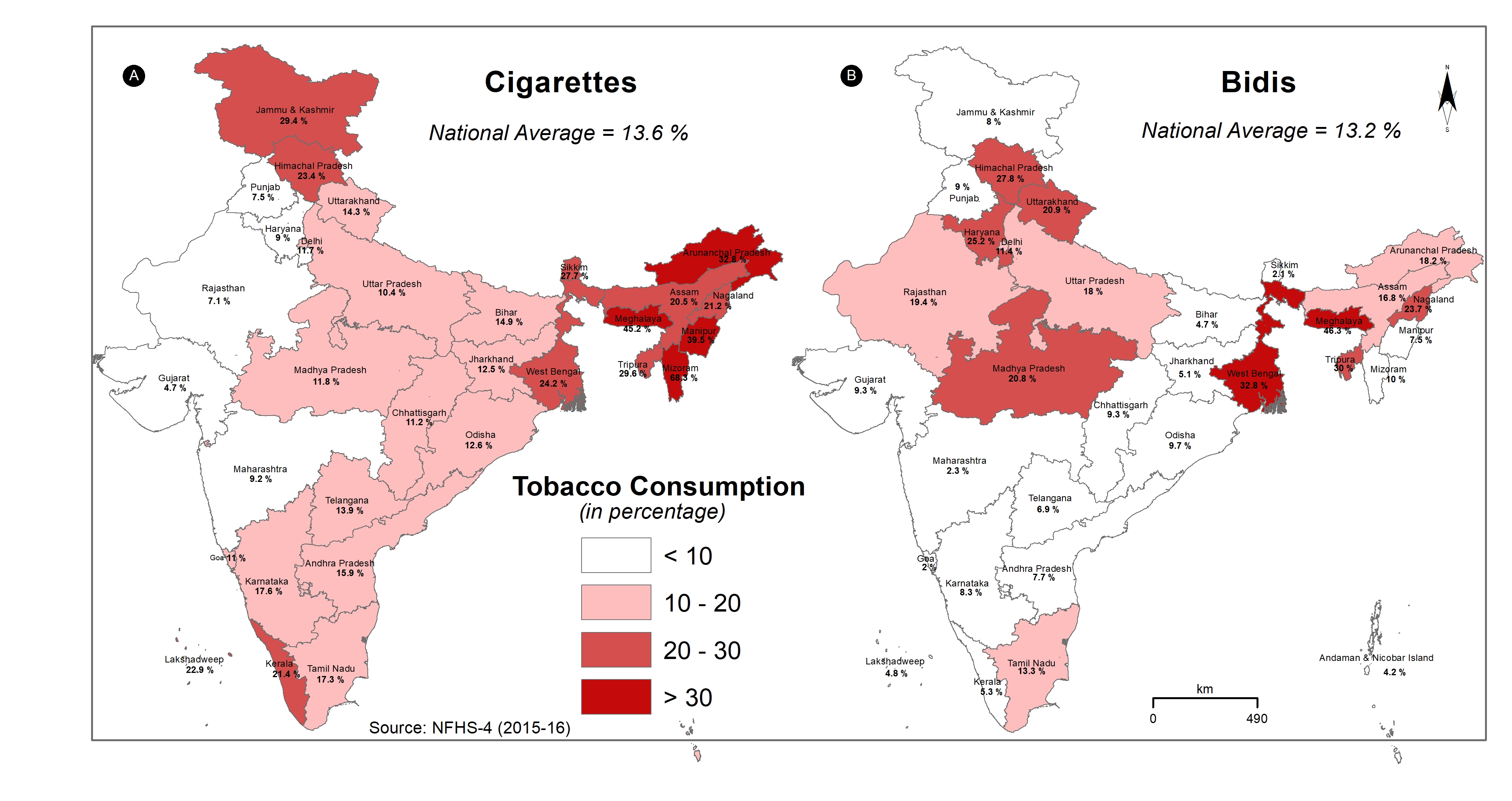

Supplement: Supplementary Figure S1 — State-wise spatial distribution of (A) cigarettes and (B) bidis (hand-rolled tobacco product) in India. The darker red shades in the maps show higher tobacco consumption. [file Image_1.JPEG]
