# Supplementary material for: Spatial epidemiology of acute respiratory infections in children under 5 years and associated risk factors in India: District-level analysis of health, household, and environmental datasets
Source: Front Public Health. 2022 Dec 13;10:906248. doi: 10.3389/fpubh.2022.906248 (PMC9792853; doi:10.3389/fpubh.2022.906248)
Supplement: Supplementary Table S1 — Descriptive statistics of the dependent and independent variables. [file Table_1.docx]

**Table S1:** **Descriptive Statistics of the dependent and independent variables (N=640 districts)**

| **Variables** | **Mean** | **SD** | **Median (p50)** | **p25** | **p75** | **min** | **max** |
| --- | --- | --- | --- | --- | --- | --- | --- |
| **ARI per 100 population** | **2.66** | **2.46** | **2.00** | **0.95** | **3.56** | **0.00** | **19.51** |
| PM 2.5 (µg/m3) | 62.49 | 30.48 | 52.60 | 39.65 | 85.75 | 0.00 | 133.2 |
| Unclean fuel use (%) | 66.37 | 23.90 | 73.68 | 51.33 | 85.87 | 0.39 | 98.7 |
| Improved sanitation (%) | 47.71 | 22.88 | 46.00 | 28.65 | 65.80 | 0.00 | 99.5 |
| Illiteracy (%) | 37.53 | 10.53 | 37.95 | 29.50 | 44.90 | 11.30 | 71.2 |
| Child immunization (%) | 61.39 | 18.72 | 62.40 | 49.20 | 74.05 | 0.00 | 100.0 |
| Rural districts | 0.70 | 0.46 | 1.00 | 0.00 | 1.00 | 0.00 | 1.0 |
| Tobacco men (%) | 47.80 | 16.16 | 49.00 | 36.15 | 58.90 | 0.00 | 100.0 |
| Tobacco women (%) | 9.63 | 12.34 | 5.20 | 2.10 | 12.20 | 0.00 | 78.1 |
| Diarrhoea in children (%) | 8.38 | 5.10 | 7.48 | 4.98 | 10.80 | 0.00 | 44.7 |
| Low birth weight (%) | 13.96 | 5.72 | 13.84 | 9.78 | 17.79 | 0.73 | 35.7 |
| Poor households (%) | 21.41 | 1.28 | 21.19 | 20.41 | 22.36 | 18.64 | 24.9 |
| Maternal BMI (mean) | 44.97 | 27.14 | 45.72 | 21.10 | 69.51 | 0.00 | 94.1 |
| Household size (mean) | 6.34 | 0.80 | 6.30 | 5.76 | 6.87 | 4.23 | 9.0 |
| Number of children (mean) | 2.42 | 0.48 | 2.34 | 2.03 | 2.76 | 1.50 | 4.0 |
| Months of breastfeeding (median) | 15.20 | 3.30 | 15.00 | 12.00 | 17.00 | 5.00 | 26.0 |

SD= standard deviation, ARI= Acute respiratory infections, p25= 25^th^ percentile, p75=75^th^ percentile, p50=50^th^ percentile or median
